# Fin-Tail Coordination during Escape and Predatory Behavior in Larval Zebrafish

**DOI:** 10.1371/journal.pone.0032295

**Published:** 2012-02-16

**Authors:** Phil McClenahan, Michael Troup, Ethan K. Scott

**Affiliations:** School of Biomedical Sciences, The University of Queensland, St. Lucia, Queensland, Australia; Institute of Marine Research, Norway

## Abstract

Larval zebrafish innately perform a suite of behaviors that are tightly linked to their evolutionary past, notably escape from threatening stimuli and pursuit and capture of prey. These behaviors have been carefully examined in the past, but mostly with regard to the movements of the trunk and tail of the larvae. Here, we employ kinematics analyses to describe the movements of the pectoral fins during escape and predatory behavior. In accord with previous studies, we find roles for the pectoral fins in slow swimming and immediately after striking prey. We find novel roles for the pectoral fins in long-latency, but not in short-latency C-bends. We also observe fin movements that occur during orienting J-turns and S-starts that drive high-velocity predatory strikes. Finally, we find that the use of pectoral fins following a predatory strike is scaled to the velocity of the strike, supporting a role for the fins in braking. The implications of these results for central control of coordinated movements are discussed, and we hope that these results will provide baselines for future analyses of cross-body coordination using mutants, morphants, and transgenic approaches.

## Introduction

Fish, in order to capture prey, avoid predators, and navigate their environments, need to coordinate movements across their bodies. This involves fluid movements of the eyes, tail, jaws, and fins. The pectoral fins are especially important for propulsion, steering, and stability, and have been shown to undergo a wide variety of motions, depending on the species and the type of movement being executed (reviewed by [1]). In labriform swimming, where propulsion is largely provided by the pectoral fins, these fins move in a manner that generates constant or near-constant propulsive force [2], [3], [4]. During other forms of swimming, they contribute to changes in pitch [5], turning [6], hovering [7], and braking [7]. While the muscular control of pectoral fins has been thoroughly studied [8], [9], [10], [11], [12], [13], and their basic innervation has been described [14], [15], [16], [17], [18], the central neural circuits controlling their movements, and the neural mechanisms by which they are coordinated with other parts of the body are less well understood. This is due in part to the fact that most research on pectoral fins has been done in an assortment of species selected for particular swimming motions or ecological niches. For practical reasons, most of these species are not well suited to laboratory rearing, genetic manipulation, or functional microscopy, and this has restricted the analyses that have been done on central control of pectoral fin movement and coordination.

Larval zebrafish (*Danio rerio*) display a small number of simple locomotor behaviors that are critical to their survival. In the absence of stimuli, larvae move using slow swims and routine turns [19]. When confronted with a threatening stimulus such as a loud noise or being touched with a probe, larvae show escape behavior, which has been described as a high-amplitude bend of the tail resulting in a change of direction followed by a rapid swim away from the site of the startle [19], [20], [21]. The fast swims during startle responses have been shown to be distinct from routine slow swims in terms of their tail kinematics. At roughly 4 days post fertilization (dpf), zebrafish larvae begin predatory behavior, in which they track, pursue, and attack prey (typically paramecia) [22], [23]. These behaviors have been extraordinarily useful for studying motor circuits, because they are simple, repeatable, and can be induced in a lab setting. Furthermore, the well-characterized hindbrain and spinal anatomy of zebrafish combined with mutants, laser ablations, and transgenic expression of proteins have permitted researchers to describe the genetic, physiological, and neural underpinnings of these motions. Notable examples include the Mauthner neurons' role in triggering startle responses [20], [24], [25], the spinal circuits mediating lateral inhibition during escape [25], [26], the identification of spinal KA neurons as the drivers of forward swimming [27], and neural control of swimming [28], [29], [30], [31].

A majority of the studies on zebrafish larval locomotion have focused on movements of the tail, and on the spinal circuits that control these movements. Less is known about the involvement of the fins in these behaviors, and the neural mechanisms by which fins are controlled and coordinated with the rest of the body are poorly understood. In zebrafish, the pectoral fins have their basic musculature [32], [33] and motor innervation [34], [35] by 5dpf. Their activity during larval forward swimming has been detailed previously. During slow swimming in larvae, the pectoral fins show coordinated alternating movements in which each fin abducts when the tail bends to the opposite side of the body, and then adducts back against the body when the tail bends ipsilaterally [36], [37], [38], [39]. These fin movements are absent during fast swims that are a part of escape behavior [38], [39]. Additionally, the pectoral fins have been implicated in braking and backing maneuvers following prey capture [23], [36], and in routine turns throughout development [40]. The involvement of the fins in startle behavior, and the intricate details of fin activity in orienting turns and predatory strike have not been explored in zebrafish.

Given the advantages that larval zebrafish provide for characterizing neural circuits [41], [42] there is a strong incentive to describe baseline behaviors in this model system. Specifically, a full description of pectoral fin-tail coordination would make it possible to explore the neural networks that underlie coordinated movement. Here, we present a method for describing the relationship between the tail and pectoral fins of zebrafish larvae, and we then use this method to describe the movements of pectoral fins across the entire range of innate larval locomotor behaviors. We find movements consistent with prior studies, including those described for slow swimming and braking, and also identify novel roles for the fins. These include coordinated movements during long-latency, but not short-latency startle responses, probable stabilizing movements during orienting turns, and graded post-capture braking movements that are scaled to the speed of the preceding strike.

## Results

### A method for measuring tail-fin coordination in larval zebrafish

**Figure 1:**
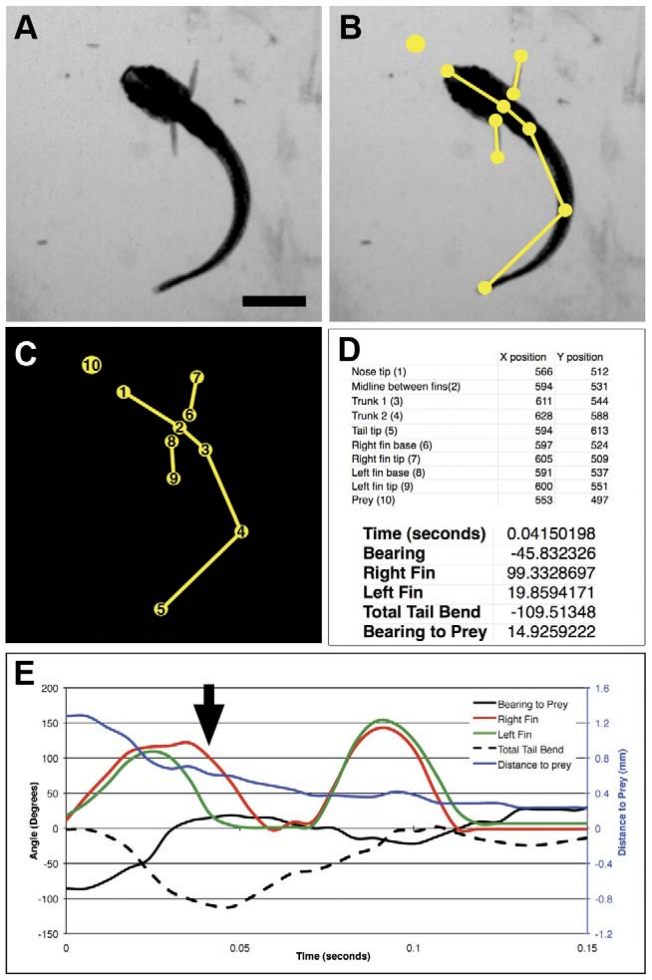
Method of kinematic analysis. Panel A shows a single frame from a high-speed movie of a 14 dpf larva that is pursuing a prey item, in this case a paramecium. In panel B, ten landmarks have been manually placed on the larva and prey. Panel C shows the resulting point-and-line representation of the larva and prey, with numbers automatically assigned to each point. The XY coordinates of each point are shown in panel D, along with automatically extracted information on the larva's fins, tail, and position relative to the paramecium. Panel E shows a portion of the overall pursuit and capture event, indicating the tail bend, extension of each fin, and distance and bearing to the paramecium through time. The arrow indicates the frame represented in panels A–D. Scale bar in A represents 1 mm.

In order to describe the movements of the head, trunk, and pectoral fins of fish, and also their relationship with prey items during capture episodes, we devised a technique involving the manual placement of anatomical landmarks followed by automated calculations of the animal's kinematics (Fig. 1). For each analyzed frame of a high-speed movie, landmarks were placed along the midline of the larva, at the base and tip of each pectoral fin, and (in the case of prey capture) on the targeted paramecium (Fig. 1A, B, and C). The XY coordinates for each of these points were automatically generated, and the resulting data on the bearing, tail bend, extension of the fins, and distance to prey were calculated (Fig. 1D). These data allowed us to map the movements of the tail and each fin, and also provided information on the larva's swim velocity, bearing to prey, and distance to prey (Fig. 1E).

### The pectoral fins in short versus long latency startle

**Figure 2:**
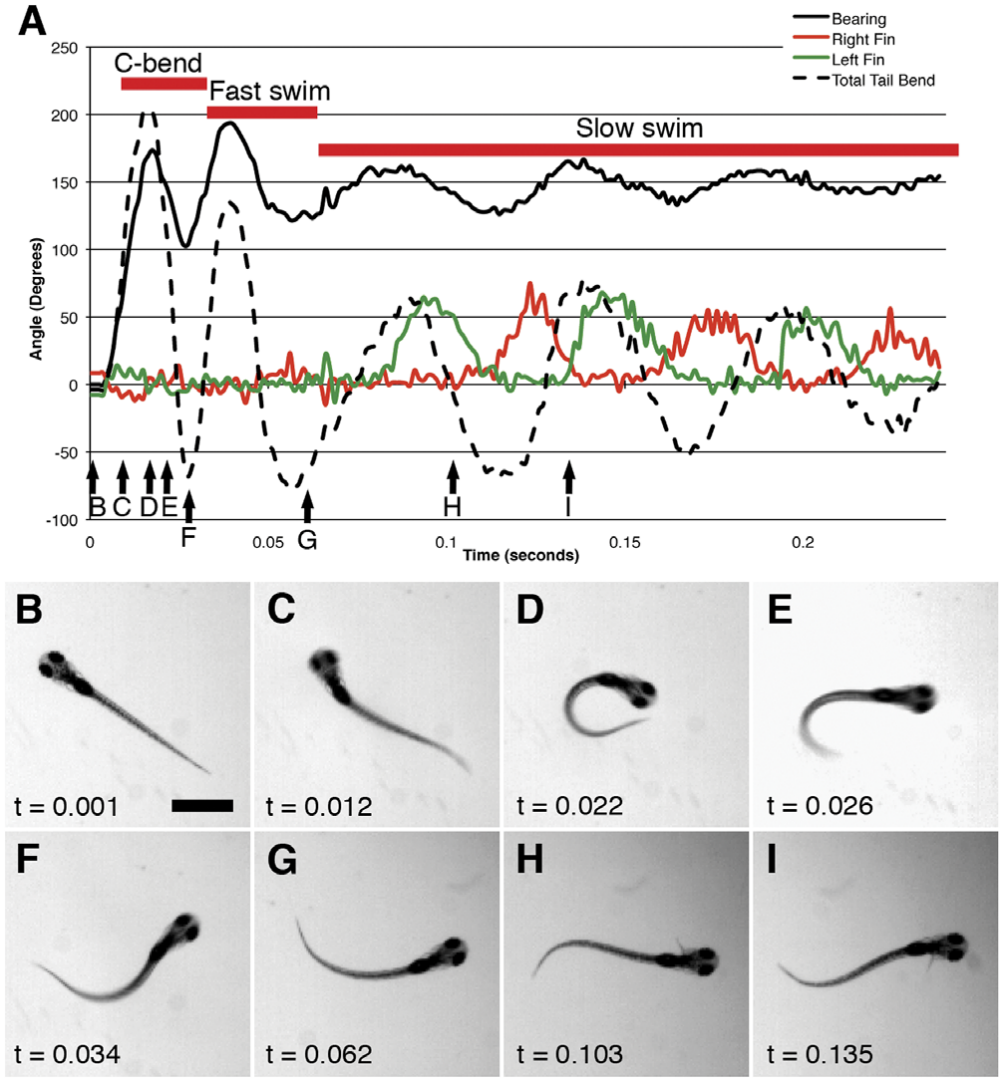
Overall structure of larval startle. The bearing (0° at t = 0), tail bend angle, and pectoral fin extensions are shown for the entirety of a response to a startling auditory stimulus (Panel A). Individual frames from the behavior are shown in Panels B–I), with accompanying timestamps. The approximate time for each of these frames is indicated by an arrow in Panel A. This startle event is composed of three phases, a rapid high-amplitude bend of the tail (C-bend), a strong counter bend and tail beat (fast swim), and then a slower, less dramatic alternating tail beat accompanied by pectoral fin extensions (slow swim). Scale bar in B represents 1 mm. The larva shown is 7 dpf.

The essential tail kinematics of startle behavior have been well described in larval zebrafish [19], [21], [43] and other species of fish [44], [45], [46]. A typical response to an acoustic or touch stimulus involves a rapid bend of the tail (C-bend), followed by a counter bend and swimming away from the site of startle. Our findings reinforce this, as illustrated by the typical startle sequence shown in Fig. 2.

Upon sensing the stimulus, the larva performs a C-bend (Fig. 2A,C,D). This results in a change in bearing of roughly 180°. This is followed by a strong counter bend in the tail (Fig. 2E,F) that partially reverses the initial bearing change. Following the bend and counter bend, the larva shows strong and alternating tail bends that propel it away from its original location (Fig. 2G). These tail bends then drop in amplitude as their frequency decreases (Fig. 2H,I). The slower, shallower tail beats are accompanied by alternating abductions of the pectoral fins, with the fin opposite the tail bend abducting while its counterpart adducts against the body (Fig. 2H,I). The kinematics of each of these responses matches those previously described for C-start [19], [24], [43], fast swimming [19], and slow swimming [37], [38], [39].

The C-bend itself has recently been shown to take two forms in larval zebrafish [43]. Larvae typically respond to intensely startling stimuli with a rapid, dramatic, and relatively consistent C-start known as a short-latency startle. Stimuli that are weaker, but still strong enough to elicit a startle often result in long-latency startle responses. These, as their name indicates, take longer to occur, and differ in other important ways [43]. Short latency startles include higher amplitude tail bend angles in the C-start, and are strikingly consistent in their kinematic details, indicating that they are an “all-or-none” response. Long-latency responses are less vigorous and more variable in their kinematics, with the magnitude of the tail bend scaling with the strength of the stimulus.

**Figure 3:**
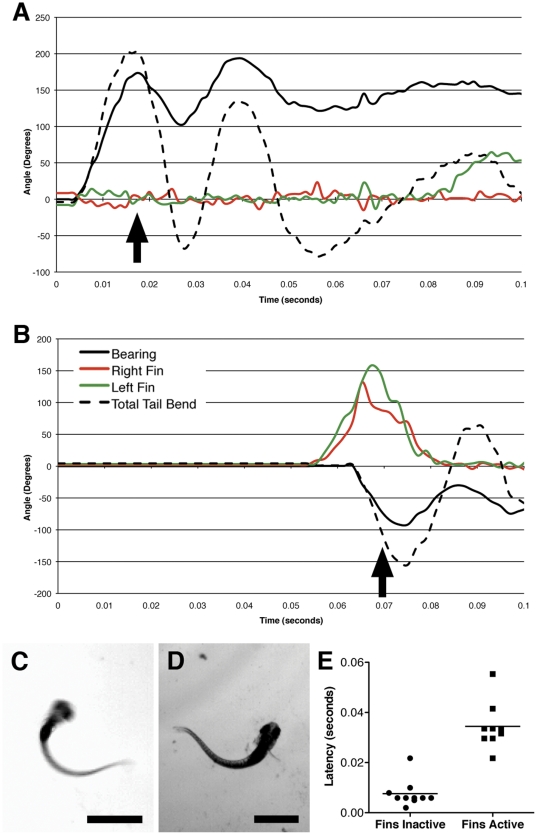
Pectoral fin involvement in long-latency, but not short-latency startle. Charts representing typical short-latency (Panel A) and long-latency (Panel B) startle responses are shown. In short-latency startle responses, the fins remain adducted during the C-bend and counter bend. Both pectoral fins are abducted and adducted in unison during the C-bend and counter bend of long-latency startles. Individual frames from a short-latency (Panel C) and a long-latency startle (Panel D) are shown, with the approximate times of the frames indicated by the arrows in Panels A and B. Panel E shows a scatter plot of startle events with and without fin involvement, and the latency to respond to the stimulus. n = 9 for responses with active fins and 10 for inactive fins, and p<0.0001 (two-tailed unpaired t-test). Scale bars in C and D indicate 1 mm. The larvae shown are 7 dpf.

We find an additional important difference between short- and long-latency responses in that short-latency C-bends occur without pectoral fin involvement, while long-latency C-bends include coordinated movements among pectoral fins and tail (Fig. 3). During short-latency startle responses, the bend and counter bend of the tail lead to changes in the direction of the larva, but the pectoral fins remain adducted throughout this stage of the behavior (Fig. 3A). In contrast, pectoral fin movements are typically a larva's first action in long-latency startles (Fig. 3B). Both fins abduct strongly, often contacting the head at their apex, while the initial C-bend occurs. They then adduct in unison to a retracted position as the counter bend in the tail occurs. This timing suggests a possible role in adding to the larva's propulsion away from the initial site where the startle occurred, or in fine-tuning the direction of the swim away from the source of the sound. These slower responses are more likely to be long-latency C-bends [43] than routine turns [20], [40], based on the maximal tail bend angles (167°+/−5°, mean +/− s.e.m.) and bearing changes (117°+/−10°, mean +/− s.e.m.) that we measured, and the fact that they arise from auditory stimuli.

### Pectoral fin kinematics of prey tracking

**Figure 4:**
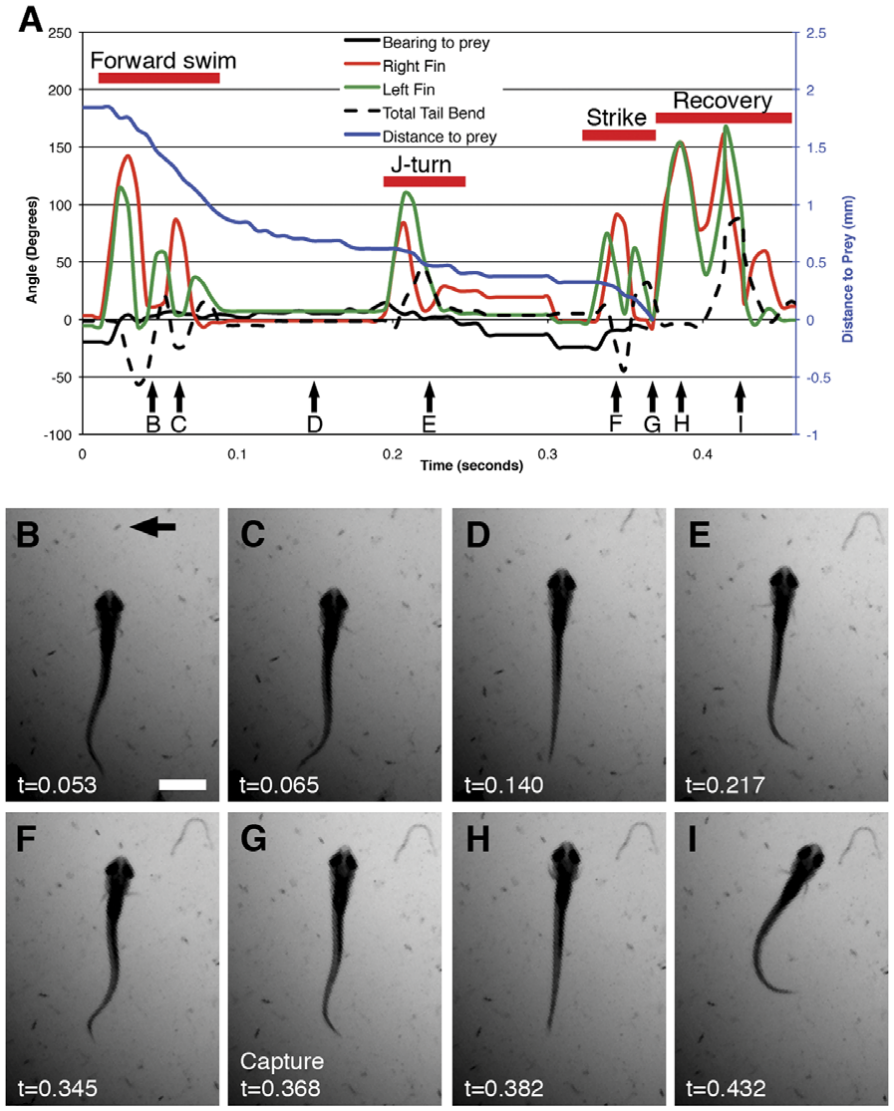
Components of prey capture. The chart in panel A shows a larva's pursuit and capture of a paramecium (arrow, B). The individual elements of the behavior are indicated by red lines. Panels B–I show individual frames from the movie of this sequence, with approximate times of the frames indicated by arrows in Panel A. The paramecium is indicated with an arrow in panel B, and all panels show the same field of view. Panels B and C show opposite tail bends of a forward swim, with extensions of the outside pectoral fin in each case. The larva pauses in panel D. Panel E shows the unilateral tail bend typical of a J-turn, with the outside pectoral fin extended. An S-bend is seen in panel F, which leads to the capture of the paramecium in panel G. Strong abductions of both pectoral fins are seen immediately after capture (Panel H), before the larva turns and swims away (Panel I). Time stamps are shown for each panel. The scale bar in B represents 1 mm. The larva shown is 7 dpf.

In order to describe more nuanced movements and maneuvers that require feedback, we observed the tail and fin movements of larvae as they tracked and attacked paramecia (Fig. 4). As has been previously described [22], [23], we found prey tracking to be composed of two operations: forward swims (Fig. 4A–C) and J-turns (Fig. 4A,E). These allowed larvae to approach and to orient toward prey, respectively, and set the stage for a predatory strike. We also observed examples in which forward swims and J-turns were apparently combined or executed simultaneously.

During forward swims, larvae showed shallow, slow tail bends, roughly equal in magnitude in the two directions. We found this to be accompanied by alternating abductions of the pectoral fins, with the fin opposite the tail bend abducted (Figs. 4A,B, and C; 5A). Forward swims resulted in an approach of the prey, but in little or no change in the bearing of the larva with regard to the prey. These kinematics resemble both previously described slow swims [37], [38], [39] and the slow swims that we observed at the end of startle responses (Fig. 2).

**Figure 5:**
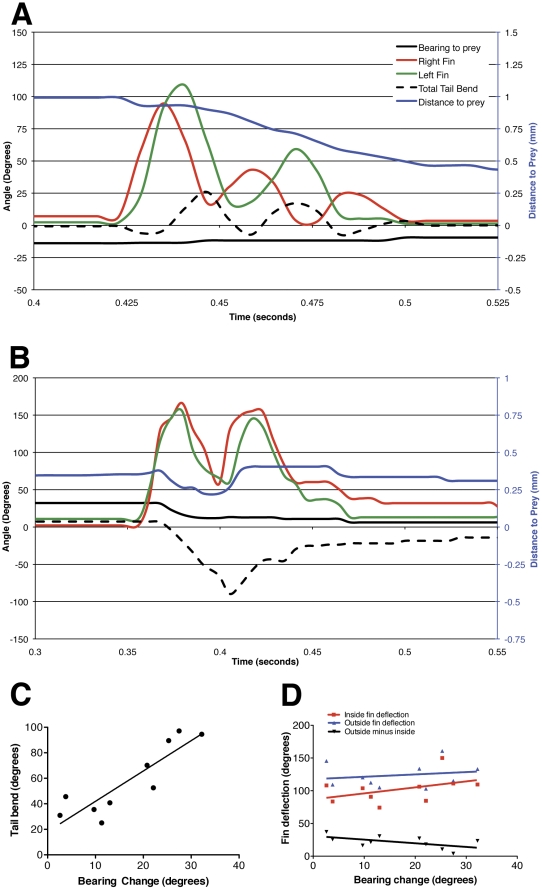
Pectoral fin movements in prey tracking. Panel A shows the kinematics of a larva swimming toward a paramecium. The forward swim takes the form of shallow, roughly symmetrical tail bends, with extension of the outside pectoral fin. This results in alternating extensions of the fins. The distance to the paramecium decreases as a result of this maneuver. A J-turn is represented in Panel B. The tail bends in a single direction (left, or negative, in this case), and the pectoral fins beat in unison. The bearing to the prey drops, but the distance to the prey remains unchanged. There is a strong relationship between the degree of the tail bend and the size of the bearing change (Panel C, p<0.001 (unpaired t-test with Welch's correction), linear regression R^2^ = 0.78), while the activities of the fins have no direct effect on the magnitude of the turn (Panel D, p>0.05 in all cases, unpaired t-test with Welch's correction).

J-turns were more variable in the kinematics of both tail and fin movements (Figs. 4A,E; 5B). As others have described [19], [22], [23], [36], J-turns involve the bending of the distal portion of the tail in one direction only, resulting in the larva's assuming a “J” shape. Consistent with other studies 19,22,23,36, we found J-turns to result in a change of bearing, generally toward the prey, but in no appreciable approach toward to the target. We found the pectoral fins to be invariably active during J-turns, but these movements were variable from animal to animal and event to event. In a majority of cases, the two pectoral fins abducted and adducted in unison during J-turns, although in some cases a single fin would move. We did not observe alternating movements of the fins during J-turns. Interestingly, the fin contralateral to the direction of the tail bend (the outside fin, hereafter) invariably (n = 18) abducted to a greater degree than the fin ipsilateral to the tail (the inside fin). Outside fins abducted to 123.8+/−6.0° (mean+/−s.e.m.), while inside fins averaged 102.2+/−6.7° (n = 10, paired t-test, p<0.0001). In some instances, the outside fin also had a greater number of movements than the inside fin.

These results do not clarify the pectoral fins' function in the execution of a J-turn. One possibility is that they are driving the change in direction, and that the greater activity from the outside fin rotates the anterior aspect of the animal's trunk in the opposite direction. Another possibility is that the tail provides the force for the change in direction, and that the fins are involved in stabilizing the larva. To identify the kinematic element that drives bearing change, we performed linear regressions of various quantitative kinematic measurements against the magnitude of the change in the animal's direction for the same maneuver. We found a strong and significant relationship between the maximal angle of the tail bend in a J-turn and the change in the animal's direction for that turn (Fig. 5C). Similar tests of the abduction of the inside fin, the abduction of the outside fin, and the differential between inside and outside fin abductions versus bearing changed failed to reveal any significant relationships (Fig. 5D). Combined, these analyses indicate that it is the J-bend in the tail, rather than movements of the pectoral fins, that drives the larva's turn.

### Predatory strike and recovery

**Figure 6:**
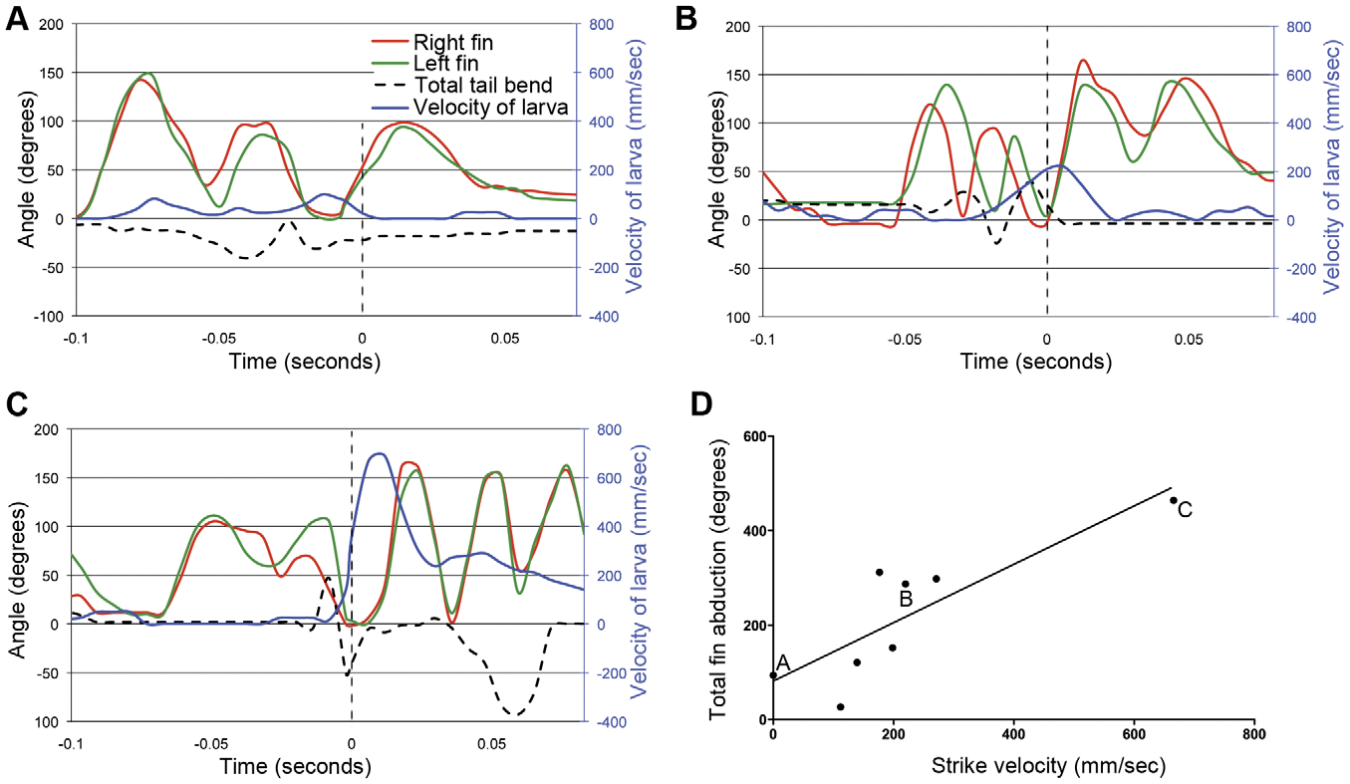
Pectoral fin abduction scales with strike velocity. Panels A, B and C show the movements of the tail and both pectoral fins for strikes of increasing velocity. The velocity of the larva is shown in blue. The moment of capture (t = 0) is indicated by a vertical dotted line. Panel D shows the correlation that exists between strike velocity and the total fin adduction that occurs following the strike (n = 8; unpaired t-test with Welch's correction, p<0.001; linear regression, R^2^ = 0.70). The events shown in panels A, B, and C are indicated.

The purpose of prey tracking is to bring the larva into position to attack the prey. Once it is close to the prey and well oriented, the larva can strike and capture it. Attacking maneuvers for fish, including zebrafish larvae, have been described as being either predominantly sucking or ram feeding motions [23], [36]. Fish that rely heavily on suction typically have little forward momentum at the time of capture, whereas those fish executing ram feeding rely on forward momentum to overtake their prey. In our study, we have observed both types of attack, but have found that most strikes combined ram feeding with suction (Fig. 6).

In some of the attacks that we observed (1/11 at 7 dpf and 4/8 at 14 dpf), the larva captured the prey using suction alone. An example is shown in Fig. 6A and Video S1. In this case, the larva sucks the paramecium into its mouth (Fig. 6A) without any forward momentum of its own. In this case, the capture event is followed by a single moderate abduction of both pectoral fins before the animal comes to rest.

More frequently (8/11 at 7 dpf and 3/8 at 14 dpf), we observed cases where forward swimming (ram feeding) and suction were combined in the capture strike (Fig. 6B and Video S2). In this representative example, the attack takes the form of repeated shallow tail beats that are accompanied by alternating extensions of the pectoral fins (Fig. 6B), as in a slow swim. The forward motion of the larva (gauged by the blue line in Fig. 6B) is more rapid than for a pure suction capture (Fig. 6A). Evidence for suction can be found in a movement of the paramecium toward the larva's mouth, and a streamlining of the paramecium into the larva's mouth at the time of capture (Video S2). Following the capture, the pectoral fins show two high-amplitude abductions.

The final type of capture that we observed (2/11 at 7 dpf and 1/8 at 14 dpf) involved an explosive forward swim and a capture of the prey reliant entirely on forward momentum (Fig. 6C and Video S3). Here, an S-bend of the tail propels the larva forward, providing velocity for the strike (blue line in Fig. 6C). Following capture, the larva extends it pectoral fins repeatedly, dramatically, and in unison.

It has been proposed that abduction of the pectoral fins after ram feeding provides braking for the fish, causing it to hold its position rather than drift forward away from the site of the strike. To test this idea, we compared the number and magnitude of pectoral fin adductions with the velocity of the larva at the moment of prey capture for all capture events performed at 7 dpf. We found a significant correlation between the larva's strike velocity and the combined angles of post-strike fin abductions (Fig. 6D). These data support the idea that the pectoral fins are used to slow the larva, since higher velocity strikes would require more braking in order to stabilize the larva's position.

## Discussion

We have observed and quantified the kinematic movements of zebrafish larvae as they execute common locomotor behaviors. The objective of the study has been to describe how the tail and pectoral fins are coordinated as these movements proceed, and how this coordination may underlie the successful completion of the larva's spatial goals. We have found pectoral fin involvement in almost all of the stereotyped movements that larvae execute, the exceptions being short-latency C-bends and fast swimming. In some cases, these results confirm and reinforce observations of pectoral fin movements seen in previous studies, such as the alternating movements of the fins during slow swimming [36], [37], [38], [39] and the extension of fins following prey capture [23], [36].

### Pectoral fins as a component of a slow, nuanced startle response

The typical startle behavior that we observed involved a C-bend followed by a fast swim, and then a slow swim. Fast swims, which are not routinely performed in the absence of stimuli, are characterized by a high temporal frequency, high angular tail bend, high velocity, and pronounced head yaw [19]. Slow swims show a lower frequency, with lower magnitude tail bends and yaw. Additionally, slow swims have been demonstrated in both zebrafish [37], [38], [39] and other species [47] to include alternating pectoral fin movements like the ones that we show in Fig. 2. In all of these respects, the startle behaviors that we observe appear to be composed of previously described C-bends and fast and slow swims.

One of the novel observations relates to larvae's varied responses to startling stimuli. When confronted with a robust auditory startle stimulus, a larva quickly responds with a high amplitude bend of the tail, and a strong counter bend that propels it away. On average, this short-latency response occurs within 5.3 msec of the stimulus [20]. The short-latency response is notable for its consistency, with only slight variations in response latency, angular velocity, bend duration, and counter bend magnitude. In contrast, long-latency startle responses take longer to initiate (28.2 msec after the stimulus, on average), and show a greater range of kinematic features [20]. Indeed, the kinematics of long-latency startles increase in magnitude with increased stimulus intensity [20]. As a consequence, short-latency startles can be viewed as all-or-none responses, while long-latency responses are graded.

Here, we add the observation that pectoral fins have a stereotyped and coordinated role in long-latency C-bends, but are not involved in short-latency responses. In long-latency startles, the pectoral fins are the first body part to respond, with coordinated abductions that precede the tail bend. These abductions become maximal as the tail bend reaches its highest angle, and the fins then adduct against the body as the counter bend occurs. The pectoral fins' lack of involvement in fast responses is consistent with the neural mechanisms underlying short-latency C-bends. These responses are mediated by the Mauthner neurons [20], which explains both the behavior's short latency and invariability. In contrast, Mauthner neurons are not needed for long-latency startles [43], which is consistent with more central neural processing. Gahtan et al. [48] have previously shown that, in the absence of Mauthner neurons, startling stimuli lead to activity in a large and distributed array of descending neurons in the hindbrain and the nucleus of the medial longitudinal fasciculus. It is likely that a subset of these active neurons indirectly mediate the movements of the pectoral fins that we observe in long-latency C-bends. The breadth of this network makes it plausible that it is controlling and tuning the timing and magnitude of muscle contractions in multiple muscle groups, which is a prerequisite for orchestrating a complex coordinated movement like the long-latency startle [49].

These observations fit well with pectoral fin involvement in long-latency, but not short-latency C-bends, and more generally with an evolutionary strategy involving two types of startle responses. Short latency startles provide a fast and dramatic response to the most threatening categories of inputs: touch and high-volume auditory stimuli. The Mauthner neurons allow for the speed of the response, but this circuit lacks the complexity necessary for graded responses, or for the recruitment of multiple motor pattern generators, as is required for the coordinated movement of different body parts. Less urgently threatening stimuli may not require as fast a response, so more central processing can take place, including a scaling of the response in proportion to the stimulus and the orchestration of motor programs for different body parts. Given the timing of pectoral fin movements, it is possible that they simply add propulsive force to the tail's counter bend. It has also been suggested that the long-latency startle may incorporate spatial information, allowing for a purposeful turn away from the sound source [20]. This suggests a possible role for the pectoral fins in fine-tuning the final bearing of the larva as it extends out of a counter bend, and into its escape swim. Further kinematic experiments incorporating a spatially-controlled auditory stimulus would be necessary to test this possible role for the fins.

### Fin involvement in prey capture

Prey tracking has been described as comprising forward swims, which move the larva toward the prey spatially, and J-turns, which allow the larva to orient toward the prey [22], [23]. We have observed both of these behaviors, as well as movements that combine both. We have found that forward swimming included alternating use of the pectoral fins, in coordination with tail movements, and that the fins are invariably but inconsistently involved in J-turns. As a larva executes a J-turn, its tail bends at the distal tip while both fins beat in unison. Although both fins are active, the one opposite the tail bend invariably abducts to a greater angle than its contralateral counterpart. We have shown that it is the magnitude of the tail bend, rather than fin activity, that predicts the rotational magnitude of the turn. This makes it unlikely that the fins are directly driving the change of the larva's bearing. Other possible functions for the fins include preventing counterrotation or backward creep as a result of the tail bend, or stabilizing the larva in some other way.

The pectoral fins have a distinct role in the recovery from a predatory strike. Almost all strikes are followed by simultaneous extension of both pectoral fins, as previously reported [23], [36]. We found the number and magnitude of these extensions to be variable. Slow strikes are often followed by a single, low amplitude abduction of the fins, or in the case of some suction-based strikes, no fin movements. Strikes that involve moderate forward movement usually include one or two strong abductions of the fins. High-speed strikes are generally followed by two or more strong fin movements. The qualitative observations held true in a quantitative analysis of strike speed versus fin usage. We found a significant positive correlation between the larvae's forward velocity at the time of the strike and the number and magnitude of pectoral fin abductions that followed. This provides support for the idea that these fin movements are used to slow the animal's forward momentum [23], [36]. The faster a larva's strike, the more dramatic the braking maneuver needs to be. This braking would allow the larva to reestablish its normal swim patterns, or to resume pursuit of prey in the event of a failed strike.

### Pectoral fin function in larval movement

Recently, Green et al [37] have explored the locomotor and physiological roles of pectoral fins in slow swimming zebrafish larvae. They found that *fgf24* morphant larvae lacking pectoral fins nonetheless swam normally. Neither swim performance nor stability was affected in these larvae. However, they showed a role for the slow-swim pectoral fin movements in drawing water across the surface of the larva, apparently to aid respiration. Bolstering this argument, normal larvae were found to execute more fin movements in oxygen-depleted water.

While the pectoral fins have a respiratory function in slow swimming, this is unlikely to be the reason for the other movements reported here. During long-latency startle, the C-bend and subsequent fast swimming transport the larva several millimeters in a fraction of a second, thoroughly mixing the water contacting the larva. During J-turns, the pectoral fins move asymmetrically, with more movements from the outside fin than the inside fin. Such asymmetry would not likely aid in respiration, but could providing a stabilizing counterbalance to the asymmetrical movements of the tail. Finally, the movements of the fins following a predatory strike are scaled with the velocity of the strike. This means that the fins are most active in the cases where the water around the larva will be mixing the most. As described above, the most obvious explanation for this result is that the fins are providing a braking force, allowing the larva to stabilize its position following the strike.

With the results presented here and elsewhere, these functions for the pectoral fins remain speculative. These predicted functions for the pectoral fins could be tested by observing the escape and prey capture performance of *fgf24* morphants lacking pectoral fins [37].

### Final thoughts and future directions

Many of the interesting coordinated movements that larval fish execute take place rapidly, and as a consequence, they can only be properly analyzed through high-speed imaging. The downside of this is that quantitative data must be extracted from a large number of frames in order to describe the movements. This, in turn, has provided a strong incentive to automate kinematic analyses. The result has been several effective programs for describing movements of the trunk and tail automatically [20], [50] or semi-automatically [38]. These automated approaches benefit from the relative clarity with which the tails of larvae can be imaged, even at high speed. Observing the pectoral (and other) fins is more difficult because they are thin, often move quickly, and disappear against the body when adducted. All of these factors complicated, and eventually prevented, our efforts to automate fin kinematic measurements. The manual system reported here is accurate but labor-intense, and a strong incentive remains to automate this process, either through improved imaging or software.

Zebrafish larvae present an appealing system for studying basic motor functions and the neural circuits that drive them. Available genetic techniques allow for the roles of specific genes to be analyzed, and transgenic approaches permit genetically encoded tools to be expressed in specified parts of circuits. As a consequence, several groups have recently used zebrafish larvae to elucidate circuits underlying forward swimming and startle [25], [26], [27], [51], [52]. These studies have, however, been restricted to the control of the trunk and tail in these behaviors, and the motor circuits controlling the fins remain less well characterized. One goal of this report is to provide kinematic descriptions and quantitative measurements of the fin activity that accompanies better-characterized tail movements. These should allow for future studies into the motor centers driving forelimb movements, and into the brain regions that coordinate and calibrate fluid motions across the body.

## Materials and Methods

### Ethics Statement

All experiments were carried out in accordance with relevant regulatory standards for animal ethics, and were approved by the University of Queensland Animal Welfare Unit.

### Fish rearing

Zebrafish (*Danio rerio*) of the Tupfel long fin (TL) strain were housed at 26°C, and fed a standard diet of live artemia. Larvae were raised in E3 media at 28.5°C, with regular water changes and cleaning. The larvae used in this study were tested at 7 or 14 dpf, with average lengths of 4.65 mm and 6.93 mm, respectively. Animals tested at 14 dpf were fed rotifers from 5 dpf until the time of testing.

### Startle and prey capture

For startle experiments, larvae were placed, 2 or 3 per dish, into 55 mm Petrie dishes in E3 media. Dishes were placed into a custom-built Plexiglas platform with a 70 mm computer-controlled speaker mounted on it. This platform was placed under a Nikon SMZ-745T dissecting microscope and illuminated from below by the microscope's halogen lamp. The startle stimulus took the form of a 550 hz tone that was applied while high-speed imaging was in progress. The categorization of startles as being fin-active or fin-inactive was done by a scorer who was blind to the latency of the startle. For prey capture, 2 or 3 larvae were placed in a 55 mm Petrie dish with roughly 100 paramecia (*Paramecium caudatum*, Southern Biological, Nunawading, Victoria, Australia) and filmed as for startle behavior.

### Image capture

Movies were taken at 506 frames per second at a resolution of 1200×1064 pixels using a Fastec HiSpec camera (Fastec Imaging, San Diego, CA, USA) mounted on a Nikon SMZ-745T dissecting microscope. Imaging began at the time of stimulus presentation for startle behavior. For prey capture, an operator triggered Fastec software upon observing a strike movement, thus saving the prior five seconds of data. Kinematics were quantified for each third frame in most cases, but less often during periods of inactivity.

### Method for quantifying fin-tail coordination

For each frame analyzed, points were manually placed at (1) the anterior-most point, (2) the midline between the base of the pectoral fins, (3) the midline between the resting points for the tips of the pectoral fins, (4) the midline halfway from the pectoral fins to the tip of the tail, and (5) the tip of the tail. These five points laid out the midline of the animal, defined the animal's bearing (from point 2 to point 1), the tail bend (sum of the angles at points 3 and 4), and provided a baseline (from point 2 to point 3) from which to calculate pectoral fin extensions. Additional points were placed at the (6) base and (7) tip of the right pectoral fin, and the (8) base and (9) tip of the left pectoral fin. These allowed the angles of the fins to be compared to the baseline angle, yielding the degree of abduction of each of the fins. Finally, for prey capture events, a point (10) was placed on the prey. This permitted the bearing and distance to the prey to be calculated.

Coordinate data were exported into Microsoft Excel, where calculations were carried out as follows. Bearing was the angle of the line from the trunk midline to the tip of the nose. A bearing of zero was defined as the bearing at t = 0, and was positive if the animal rotated clockwise and negative if counterclockwise. For prey capture, bearing was the angle between the larva's bearing and the true bearing to the prey, and was positive if the prey was to the larva's right, negative if to the left. Tail bend was defined as the sum of the angles formed at the two most posterior angles of the schematic larva (positive for a right bend, negative for a left bend). Fin extension was the absolute value of the difference between the angle of the fin and the angle of the trunk where the fin would normally lie flat. Macros for ImageJ and templates for Excel will be provided upon request.

To quantify strike velocity, the distance travelled by the larva during seven frames of the high-speed movie spanning the capture were divided by 14 ms, the period of elapsed time during these frames. Total fin abduction following a strike was quantified by averaging the left and right maximal fin abductions for each fin movement, and then summing the averages if more than one fin movement followed the strike.

A table (Table S1) of mean values for the data presented in this paper is available as supplementary material.

## Supporting Information

Table S1 **Mean data.** The mean values for measurements taken in this study are indicated ± standard deviation. Experimental n is indicated in parentheses.
(DOC)

Video S1 **Movie of the predatory strike represented in** **Fig. 6A** **.** The paramecium is captured solely by suction, and there is no forward movement from the larva at the time of capture. The pectoral fins adduct moderately after the capture. The movie plays at 1/10 real speed.
(MOV)

Video S2 **Movie of the predatory strike represented in** **Fig. 6B** **.** The paramecium is captured by a combination of suction and ram-feeding. There are two high-amplitude adductions of the pectoral fins following the capture. The movie plays at 1/10 real speed.
(MOV)

Video S3 **Movie of the predatory strike represented in** **Fig. 6C** **.** The paramecium is captured by an explosive forward swim resulting from an S-start. The pectoral fins show three strong adductions after the paramecium is captured. The movie plays at 1/10 real speed.
(MOV)
