# Supplementary material for: Fin-Tail Coordination during Escape and Predatory Behavior in Larval Zebrafish
Source: PLoS One. 2012 Feb 16;7(2):e32295. doi: 10.1371/journal.pone.0032295 (PMC3281131; doi:10.1371/journal.pone.0032295)
Supplement: Table S1 — Mean data. The mean values for measurements taken in this study are indicated ± standard deviation. Experimental n is indicated in parentheses. (DOC) [file pone.0032295.s001.doc]

|  | 7dpf | 14dpf |
| --- | --- | --- |
| Length (mm) | 4.65 (0.32) | 6.93 (0.51) |
| **Short Latency Startle** | | |
| Latency to first response (ms) | 7.6±5.4 (10) | N/A |
| Peak tail bend angle (degrees) | 191.6±35.5 (10) | N/A |
| Latency to peak tail bend (ms) | 19.4±6.7 (10) | N/A |
| Peak pectoral fin abduction, average of both fins (degrees) | 10.9±7.6 (10) | N/A |
| **Long Latency Startle** | | |
| Latency to first response (ms) | 34.5±9.4 (9) | 55.8±12.7 (8) |
| Peak tail bend angle (degrees) | 167.3±8.2 (9) | 166.2±14.2 (9) |
| Latency to peak tail bend (ms) | 46.7±12.5 (8) | 84.2±15.6 (8) |
| Peak pectoral fin abduction, average of both fins (degrees) | 88.1±44.5 (9) | 151.2±29.7 (9) |
| **J-turn** | | |
| Tail bend angle (degrees) | 58.2±27.5 (10) | 60.9±33.3 (8) |
| Change in bearing (degrees) | 16.8±10.2 (10) | 19.2±13.7 (8) |
| Inside fin abduction (degrees) | 102.2±21.2 (10) | 89.2±20.6 (8) |
| Outside fin abduction (degrees) | 123.8±18.9 (10) | 124.7±17.9 (8) |
| **Slow Swim** | | |
| Peak angle of tail bend (degrees) | 21.3±16.2 (17) | 17.3±15.4 (7) |
| Inside fin abduction (degrees) | 10.8±19.4 (17) | 5.0±6.5 (7) |
| Outside fin abduction (degrees) | 74.1±28.7 (17) | 74.8±25.4 (7) |
